# Augmented Diagnostic Accuracy of Ultrasonography for Diagnosing Carpal Tunnel Syndrome Using an Optimised Wrist Circumference-Dependent Cross-Sectional Area Equation

**DOI:** 10.3389/fneur.2020.577052

**Published:** 2020-09-25

**Authors:** Tom B. G. Olde Dubbelink, Floriaan G. C. M. De Kleermaeker, Jan Meulstee, Ronald H. M. A. Bartels, Franka Claes, Wim I. M. Verhagen

**Affiliations:** ^1^Department of Neurology, Canisius-Wilhelmina Hospital, Nijmegen, Netherlands; ^2^Department of Neurology, VieCuri Hospital, Venlo, Netherlands; ^3^Department of Neurosurgery, Radboud University Medical Centre, Nijmegen, Netherlands; ^4^Department of Neurology, Vlietland Hospital, Schiedam, Netherlands

**Keywords:** carpal tunnel syndrome (CTS), ultrasonography, diagnostics, cross-sectional area (CSA), median nerve, wrist circumference

## Abstract

**Introduction:** In diagnosing carpal tunnel syndrome (CTS) there is no consensus about the upper limit of normal (ULN) of the cross-sectional area (CSA) of the median nerve at the carpal tunnel inlet. A previous study showed wrist circumference is the most important independent predictor for the ULN. In this study we optimised a wrist circumference-dependent ULN equation for optimal diagnostic accuracy and compared it to the generally used fixed ULN of 11 mm^2^.

**Methods:** CSA and wrist circumference were measured in a prospective cohort of 253 patients (clinically defined CTS) and 96 healthy controls. An equation for the ULN for CSA was developed by means of univariable regression analysis. We calculated *z*-scores for all patients and healthy controls, and analysed these scores in a ROC curve and a decision plot. Sensitivity and specificity were determined and compared to fixed ULN values.

**Results:** We found augmented diagnostic accuracy of our newly developed equation y = 0.88 ^*^ x −4.0, where y = the ULN of the CSA and x = wrist circumference. This equation has a corresponding sensitivity and specificity of 75% compared to a sensitivity of 70% while using a fixed cut-off value of 11 mm^2^ (*p* = 0.015).

**Conclusion:** Optimising the regression equation for wrist circumference-dependent ULN cross-sectional area of the median nerve at the wrist inlet might improve diagnostic accuracy of ultrasonography in patients with carpal tunnel syndrome and seems to be more accurate than using fixed cut-off values.

## Introduction

Carpal tunnel syndrome (CTS) can be diagnosed by taking accurate medical history in combination with clinical assessment (1). Ultrasonography (US) is the most commonly used test after electrodiagnostic testing (EDX), in confirming the clinical diagnosis of CTS. Especially when surgical decompression is considered, EDX or ultrasonography assessment is performed for confirming the diagnosis. The AAOS Clinical Guidelines recommend EDX testing for CTS patients when surgery is being considered (2) while the Dutch consensus CTS guideline states no additional studies are needed in case of classical CTS (3). An earlier study showed that only a minority of surgeons would perform surgery without electrodiagnostic confirmation of CTS (4).

For ultrasonography, alteration of the shape of the median nerve is evaluated and an enlarged cross-sectional area (CSA) of the median nerve at the carpel tunnel is frequently used to confirm CTS diagnosis (5). Currently, using ultrasonographic evaluation of increase in size of the median nerve at the carpal tunnel, fixed values for the upper limit of normal (ULN) with a broad range of 8.5–15 mm^2^ are reported (6–14). This broad range of the normal values may be affected by morphometric factors, as well as age and sex as described in the literature (15–18). An earlier study reported similar diagnostic accuracy of sonography to that for EDX studies (11). Because of comparable sensitivity and patient-friendliness, ultrasonography is recommended as the first line diagnostic test for CTS in The Netherlands.

There is, however, no consensus about the upper limit of normal of the CSA of the median nerve at the carpal tunnel inlet. We previously showed a strong correlation between wrist circumference and CSA of the median nerve at the carpal tunnel inlet in subjects without signs or symptoms of carpal tunnel syndrome (15). Furthermore, we developed an equation for the ULN of the CSA which has a relatively low sensitivity (53.4%) but a very high specificity (95%) (19). It is the low sensitivity that hampers the clinical applicability of this equation. We hypothesize that, by optimising the sensitivity and specificity of this equation, an individualised upper limit of normal of the CSA based on wrist-circumference has an higher sensitivity than a fixed upper limit of normal does. We analysed a decision plot based on a receiver operating characteristic (ROC) curve of the healthy controls to augment diagnostic accuracy and we compared this to the generally used fixed ULN of 11 mm^2^ (11).

## Materials and Methods

### Methods

We prospectively enrolled patients and healthy control subjects in this observational study. We obtained written informed consent from each patient and healthy control. Approval from the local Medical Ethics Committee was obtained.

### Study Population and Sonography Assessment

We recruited 96 healthy control subjects without signs and symptoms of CTS. All 96 healthy controls underwent medical history taking and physical examination (WV, FC). Controls with a history of diabetes mellitus, rheumatoid arthritis, wrist trauma or BMI >35 kg/m^2^ were excluded. Controls with bifid median nerves were excludes as well. Both wrists were measured, we randomly included only one wrist and CSA because we did not find any differences in earlier studies (15).

A total of 253 clinically defined CTS patients were included if they had pain and/or paraesthesia in the territory innervated by the median nerve. Two or more of the following clinical CTS criteria had to be fulfilled: (1) nocturnal paraesthesia, (2) aggravation of paraesthesia by activities such as driving a car, riding a bike, holding a book, or holding a telephone, (3) positive Flick sign (paraesthesia relieved by shaking the affected hand). For patients with bilateral CTS only the most severely affected hand was included.

Exclusion criteria were age under 18; history or clinical signs of polyneuropathy or known hereditary neuropathy with liability to pressure palsies; previous trauma or surgery to the wrist; history of rheumatoid arthritis; diabetes mellitus; thyroid disease; alcoholism; arthrosis of the wrist; pregnancy; severe atrophy of the abductor pollicis brevis muscle; bifid median nerve or significant language barrier.

We measured weight, height and wrist circumference at the level of the distal wrist crease using plastic measuring tape. CSA of all subjects was measured at the inlet of the carpal tunnel (Philips Diagnostic Ultrasound System model iU22, 5–17-MHz linear transducer) using the direct trace method. Electrodiagnostic technicians took the measurements, and patients underwent US and EDX according to the protocol of our previous study (15, 20). The used US parameters were: frequency 17 MHz, acoustic power 100%, dynamic range 77 dB, deepness 1.5 cm, focus position 2 cm, gain 80. US was performed by two experienced US technicians, EDX studies by a clinical neurophysiologist (JM). The cross-sectional area of the median nerve was determined by outlining the nerve contour using the inner margin of the hyperechoic rim. The CSA was calculated by the area measurement software (continuous contour trace) of the ultrasound system, rounding all measurements to the nearest 0.01 cm^2^. The mean of three separate measurements was taken as CSA at the inlet of the carpal tunnel.

### Z-score

We calculated a Z-score specific for an individual wrist circumference according to:

$$\begin{array}{l} \text{z}-\text{score}=(\text{X}-\text{μ})\text{/}\sigma =(\text{CSA measured}\\ \;-\text{CSA expected from wrist circumference})\text{/standard deviation}. \end{array}$$

X being the mean of three actual CSA measurements, μ the expected CSA calculated from the wrist circumference and σ the standard deviation.

Ergo:

$$\begin{array}{l} \text{z}-\text{score}*\text{standard deviation}=(\text{CSA measured}\\ \;-\text{CSA expected from wrist circumference}). \end{array}$$

This enables us to generate a “new” upper limit of normal based on wrist circumference (maximum CSA expected from wrist circumference).

### Statistics

Statistical analysis was performed using SPSS Statistics 26.0. Baseline characteristic for healthy controls and patients were described as mean ± SD and frequency (%). Unpaired *T*-tests were used for continuous variables with normal distribution, and the Mann–Whitney test in case of non-normal distribution for group comparisons of baseline data. McNemar's test was used for paired categorical data. We used univariable regression analyses to create equations for the ULN for CSA. We used *z*-scores, a receiver operating characteristic (ROC) curve and a decision plot to develop a new equation with optimal diagnostic accuracy. Normal distribution of data was assessed visually by plotting a histogram, using a Q–Q plot and the Kolmogorov–Smirnov test. The level of significance was set at 0.05 for all analyses.

## Results

In Table 1 the characteristics of the included healthy controls and patients are presented. The data of the 96 healthy controls were normally distributed. In the healthy controls the mean CSA was significantly smaller in women (*n* = 49); 8.8 mm^2^ (SD 1.9), compared to 10.2 mm^2^ (SD 1.9) in men (*n* = 47; *p* = 0.001).

**Table 1:** Healthy controls and patient characteristics (standard deviation between brackets).

	**Healthy controls**	**Patients**	***p***
Participants (*n*)	96	253	
Men/women	47/49	51/202	<0.001
Mean age (y)	44.6 (±11.4)	47.1 (±10.9)	0.060
Median age (y)	46	48	
Left/right	48/48	149/104	
Mean height (cm)	175.8 (±9.0)	167.3 (±7.9)	<0.001
Mean weight (kg)	77.3 (±13.5)	76.6 (±15.6)	0.692
Mean BMI	25 (±3.6)	27.3 (±4.9)	<0.001
Mean wrist circumference (cm)	16.8 (±1.4)	16.6 (±1.2)	0.293
Mean CSA (mm^2^)	9.5 (±2.0)	13.5 (±4.4)	<0.001

253 patients were consecutively enrolled in our study. In the patient group mean CSA was 13.4 mm^2^ (SD 4.4) in women (*n* = 202) and 14.0 mm^2^ (SD 4.1) in men (*n* = 51) with no statistically significant difference (*p* = 0.132).

**Figure 1:**
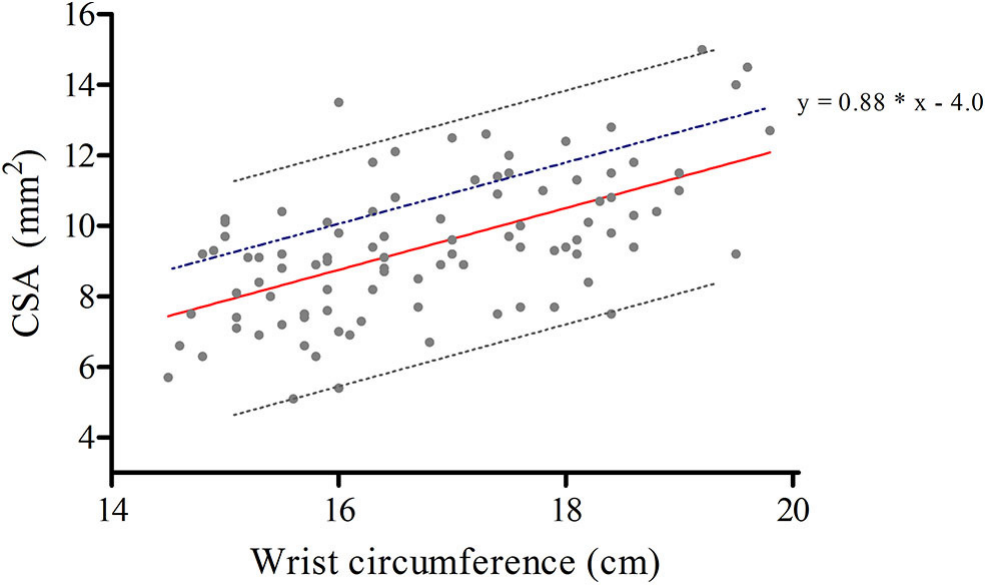
Regression analysis of the 96 healthy controls for determining the upper limit of normal (ULN) of the cross-sectional area depending on wrist circumference. Regression equations for median values (0.88 * x −5.25, red line), and for the upper limit of normal (0.88 * x −2.0, grey upper dashed line); x being the wrist circumference. The blue dashed line represents our optimised equation for the new ULN (y = 0.88 * x −4.0), adding 1.24 for each y.

Figure 1 is a scatter plot showing the results of the regression analysis of the 96 healthy controls for determining the upper limit of normal of the CSA depending on wrist circumference. The values for expected CSA, μ, were calculated by filling in the wrist circumference in the regression equation for median values. This was used to determine the *Z*-scores for all patients and healthy controls. The difference between the upper limit of normal (upper grey dashed) line and the median value (red) line is 3.25 and equals 1.96 times the standard deviation.

**Figure 2:**
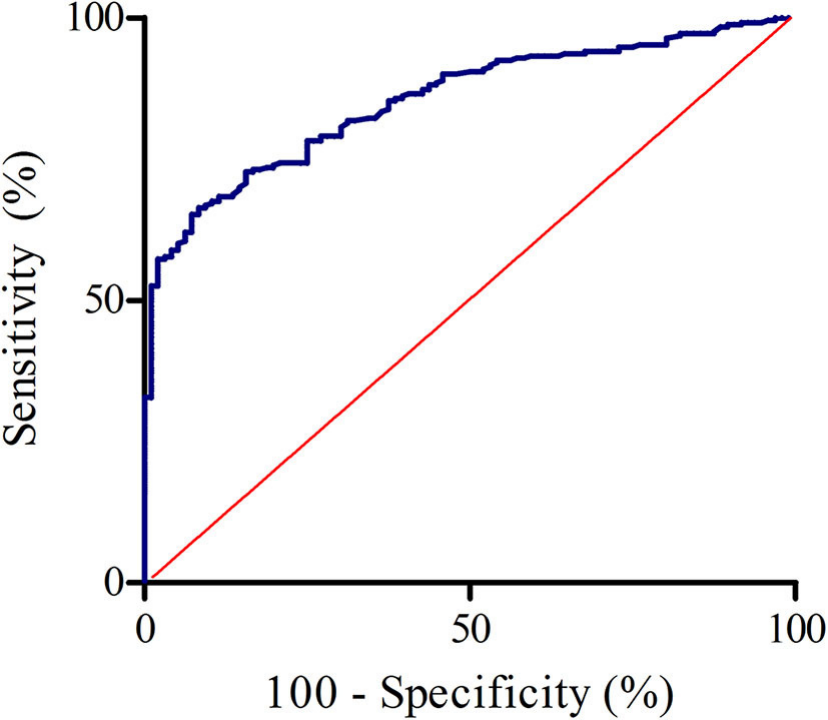
ROC curve of the calculated *z*-scores (healthy controls and clinically defined carpal tunnel syndrome patients). AUC = 0.854, 95% confidence interval: 0.815–0.893.

**Figure 3:**
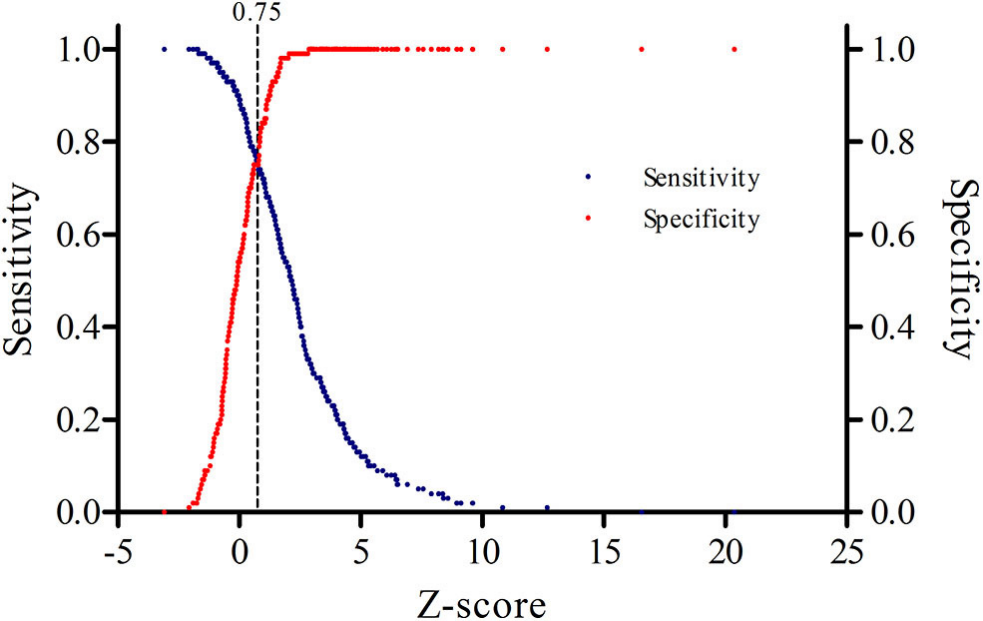
Decision plot showing that a *z*-score of 0.75 (dotted vertical line) represents the optimal cut-off point for the highest sensitivity and specificity.

By plotting the earlier mentioned *Z*-scores of all individuals (patients and healthy controls) in a receiver operating characteristic (ROC) curve we can determine the optimal coefficients for the formula for the ULN of wrist circumference-dependent CSA (Figure 2). To help determine the optimum decision level (maximum number of CTS patients correctly diagnosed as positive by ultrasonography in relation to maximum number of healthy controls correctly diagnosed as negative) we made a decision plot (Figure 3).

The discrimination between healthy controls and patients with clinically defined CTS is optimal when using a *z*-score of 0.75, as can be seen in Figure 3. Rewriting and filling in:

$$\begin{array}{l} \text{z}-\text{score}*\text{standard deviation}=(\text{CSA measured}\\ \;-\text{maximum CSA expected from wrist circumference}), \end{array}$$

results in

$$\begin{array}{l} \text{maximum CSA expected from wrist circumference}\\ \;=\text{CSA measured}-\left(\text{0}.\text{75}*\text{1}.\text{66}\right)\\ \;=\text{CSA measured}-\text{1}.\text{24}. \end{array}$$

Accordingly, the maximum wrist circumference related CSA is 1.24 higher than previously calculated while using y = 0.88 ^*^ x −5.25. This means that we have to add 1.24 for each y. As a result, and taking into account rounding errors, the optimised equation becomes y = 0.88 ^*^ x −4.0. At this *z*-value, the corresponding sensitivity is 75% and the specificity for this optimised equation is 75%.

**Figure 4:**
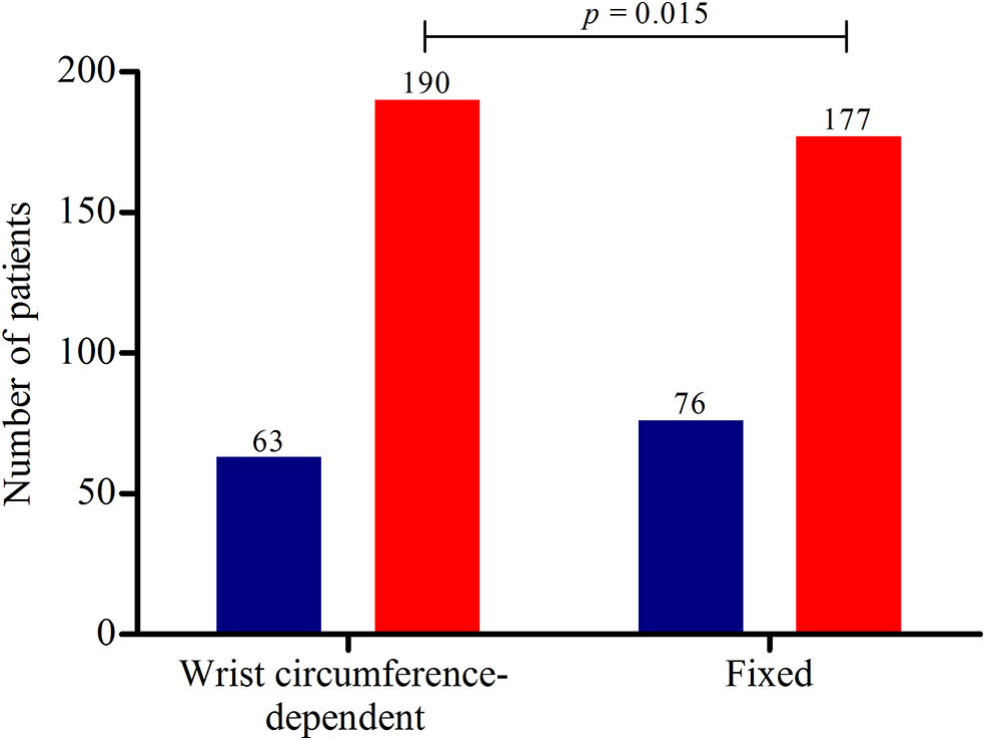
Ultrasound results of the 253 patients. The red bars represent the number of patients with abnormal ultrasonography results and the blue bars normal ultrasonography results. Using a wrist-circumference dependent upper limit of normal (ULN), 190 patients (75.1%) had abnormal US results vs. 177 patients (70.0%) using a fixed ULN of the cross-sectional area of the median nerve of 11 mm^2^ (*p* = 0.015). The mean CSA of these 190 and 177 patients with abnormal US results were 14.9 (±4.1) and 15.2 (±4.1), respectively, without a statistically significant difference (*p* = 0.201).

When applying the wrist circumference-dependent CSA equation and the aforementioned fixed cut-off value in the group of patients, we found the results as presented in Figure 4. Abnormal US results were found in 177 (70.0%) patients while using a fixed cut-off value of 11 mm^2^ compared to 190 (75.1%) patients when applying our optimised equation. The ultrasound was considered abnormal most often in the wrist circumference-dependent upper limit of normal formula group, with a statistically significant difference compared to the general fixed ULN (*p* = 0.015).

Furthermore, 54/253 (21.3%) of the clinically defined CTS patients had normal EDX results. Eighteen of these 54 (33.3%) patients had an ULN >11 mm^2^. Using our new equation (y = 0.88 ^*^ x −4.0), 23 of the 54 (42.6%) CTS patients with normal EDX results, would have an abnormal US result.

## Discussion

We found that our new equation, y = 0.88 ^*^ x −4.0 (x = wrist circumference in centimetres), has a corresponding sensitivity of 75% and a specificity of 75%. As presented in Figure 4, the sensitivity of our optimised equation is higher than this fixed cut-off value (75.1 vs. 70.0%, *p* = 0.015). By using the data from the ROC approach, increasing sensitivity and decreasing the specificity, it seems that our simple optimised equation is more valuable in daily practice when determining the upper limit of normal of the CSA at the wrist inlet and confirming the clinical suspicion of CTS. Accordingly, wrist circumference-dependent CSA upper limits of normal could be used for better diagnostic accuracy.

Diagnostic accuracy of US in CTS patients depends on the upper limit of normal of the measured CSA of the median nerve at the carpal tunnel inlet. We previously showed, by performing multivariable linear regression, that wrist circumference is the most important independent predicting factor for CSA (15), accounting for 37% of the variation in this parameter (19). We also showed an equation for the ULN of the CSA with a relatively low sensitivity but a very high specificity. In this study we improved the diagnostic accuracy of this equation in order to use US in CTS patients as a screening test. The sensitivity and specificity are rather low at 75% but in line with literature where sensitivity ranges mostly from 70–88% and specificity from 57–97% (21, 22). Earlier studies showed high positive predictive values for ultrasonography: if ultrasonography is abnormal, EDX studies were abnormal in 96.7–100% (20, 23). In daily practice, taking in account the lower costs of ultrasonography and patient-friendliness, we would suggest to perform ultrasonography as the first diagnostic test in conforming the diagnosis CTS. If ultrasonography is normal, EDX studies could be used as second diagnostic test if clinical suspicion of CTS still exists.

The additional value of ultrasonography compared to EDX studies includes detection of anomalies such as structural abnormalities at the wrist, bifid median nerves and persistent median arteries (24). As stated before US is less time consuming and more comfortable (pain-free) (25). However, not all patients with CTS do have an enlarged median nerve, maybe in part due to a short duration of symptoms (11). EDX studies can quantify nerve damage, have high sensitivity (26) and can be used to differentiate CTS from more proximal median nerve neuropathy or other conditions, for example a C6/C7 radiculopathy.

There are several limitations to our study that should be addressed. First of all, we included CTS patients with sensory symptoms in the fifth finger. We ruled out an ulnar neuropathy in these patients by medical history only, not by nerve conduction studies. However, as stated in a previous study, symptoms in the fifth finger are often reported by CTS patients, and treatment outcome does not differ compared to CTS patients with a classic presentation (27). Secondly, the electrodiagnostic technicians who performed ultrasonography investigating the healthy controls were not blinded. They may have anticipated to find no enlargement of the median nerve and this possibly influenced the measurements. The controls did not have any signs or symptoms of CTS; however, this does not always imply a normal ultrasonography result. This may have influenced the measurements the other way around. We did not calculate intra- and interobserver agreement of the CSA measurements of the median nerves in this study but we found good agreement in an earlier study (15). Furthermore, we excluded healthy controls with a BMI > 35kg/m^2^ for a representative univariable regression analysis. We hypothesised that including obese controls could result in a higher ULN of the CSA, leading to more false negatives. 14 of the 253 (5.5%) patients had a BMI >35 kg/m^2^ with the expected significantly higher wrist circumference (mean 17.5, *p* = 0.019) but non-significantly higher CSA (mean 14.9, *p* = 0.397) compared to the other patients. In addition, we excluded patients and healthy controls with bifid median nerves so our equation is not valid for these patients.

In the future, evaluating our optimised equation in populations with other morphometric features and less specific populations (not fulfilling all criteria that we used, but a clinical suspicion of CTS) would be interesting, as well as comparing this equation with other parameters, for example an intraneural flow related upper limit of normal (28). In literature, numerous possible ultrasonography parameters have been investigated. The cross-sectional area of the median nerve remains the best single criterion (5) and seems to be related with neurophysiological severity (29). The diagnostic value of wrist median nerve CSA vs. wrist-to-forearm ratio showed inconsistent results in literature (30, 31). A cross-sectional study published in 2019 by Chang et al. suggests the ulnar nerve compared to the median nerve at wrist level could serve as internal control by using median-to-ulnar-nerve difference instead of the median-to-ulnar-nerve ratio (32).

To conclude, this study shows that optimising the regression equation for wrist circumference-dependent ULN cross-sectional area of the median nerve at the wrist by fine tuning its coefficients by ROC curve analysis inlet might improve diagnostic accuracy of ultrasonography in patients with carpal tunnel syndrome and seems to be more accurate than using fixed cut-off values.

## Data Availability Statement

The data analyzed in this study is subject to the following licenses/restrictions: Offline dataset. SPSS dataset are available upon request. Requests to access these datasets should be directed to Tom B. G. Olde Dubbelink, t.oldedubbelink@cwz.nl.

## Ethics Statement

The studies involving human participants were reviewed and approved by Commissie Mensgebonden Onderzoek regio Arnhem-Nijmegen. The patients/participants provided their written informed consent to participate in this study.

## Author Contributions

TO, FK, JM, FC, and WV contributed conception and design of the study. TO and FK organised the database and performed statistical analysis. TO wrote the first draft of the manuscript. TO, FK, JM, WV, and RB wrote sections of the manuscript. All authors contributed to the manuscript revision and read and approved the final version.

## Conflict of Interest

The authors declare that the research was conducted in the absence of any commercial or financial relationships that could be construed as a potential conflict of interest.

## Abbreviations

CTS: carpal tunnel syndrome
CSA: cross-sectional area
ULN: upper limit of normal
US: ultrasonography.
